# Progress in Pathogenesis of Proteinuria

**DOI:** 10.1155/2012/314251

**Published:** 2012-05-24

**Authors:** Aihua Zhang, Songming Huang

**Affiliations:** Department of Nephrology, Nanjing Children's Hospital, Nanjing Medical University, Nanjing 210008, China

## Abstract

*Aims*. Proteinuria not only is a sign of kidney damage, but also is involved in the progression of renal diseases as an independent pathologic factor. Clinically, glomerular proteinuria is most commonly observed, which relates to structural and functional anomalies in the glomerular filtration barrier. The aim of this paper was to describe the pathogenesis of glomerular proteinuria. *Data Sources*. Articles on glomerular proteinuria retrieved from Pubmed and MEDLINE in the recent 5 years were reviewed. *Results*. The new understanding of the roles of glomerular endothelial cells and the glomerular basement membrane (GBM) in the pathogenesis of glomerular proteinuria was gained. The close relationships of slit diaphragm (SD) molecules such as nephrin, podocin, CD2-associated protein (CD2AP), a-actinin-4, transient receptor potential cation channel 6 (TRPC6), Densin and membrane-associated guanylate kinase inverted 1 (MAGI-1), *α* 3 *β* 1 integrin, WT1, phospholipase C epsilon-1 (PLCE1), Lmx1b, and MYH9, and mitochondrial disorders and circulating factors in the pathogenesis of glomerular proteinuria were also gradually discovered. *Conclusion*. Renal proteinuria is a manifestation of glomerular filtration barrier dysfunction. Not only glomerular endothelial cells and GBM, but also the glomerular podocytes and their SDs play an important role in the pathogenesis of glomerular proteinuria.

## 1. Introduction

Under normal conditions, high molecular weight proteins in the plasma (e.g., albumin and globulin) cannot pass through the filtration membrane due to the effects of the size barrier and charge barrier of the glomerular capillary filtration membrane. Low molecular weight proteins (e.g., *β*2-microglobulin (*β*2-M), *α*1-microglobulin (*α*1-M), and lysozyme), however, can freely pass through the filtration membrane, although the filtration amount is low and 95% of these proteins are reabsorbed when entering the proximal convoluted tubule. The final urine protein content is therefore low (only 30–130 mg/24 h) and consists primarily of plasma albumin (40%), immunoglobulin fragments (15%), other plasma proteins (5%), and urinary system-originating tissue proteins (40%). The protein concentration in a random urine sample is 0–80 mg/L, and the results of qualitative tests for urokinase protein are typically negative. When the urine protein exceeds 150 mg/24 h or the concentration is above 100 mg/L, the result for the qualitative protein test becomes positive. This is known as proteinuria [1, 2].

Proteinuria is the most common manifestation of renal diseases. Reviews of a number of experimental studies have shown that proteinuria is not only a sign of kidney damage, but also participates in the progression of renal diseases as an independent pathologic factor [3–5]. Clinically, glomerular proteinuria is most commonly observed and relates to structural and functional anomalies in the glomerular filtration barrier. In recent years, researchers have not only gained a new understanding of the roles of glomerular endothelial cells and the glomerular basement membrane (GBM) in the pathogenesis of proteinuria, but have also gradually discovered the close relationships of slit diaphragm (SD) molecules such as nephrin, podocin, CD2-associated protein (CD2AP), a-actinin-4, transient receptor potential cation channel 6 (TRPC6), Densin, and membrane-associated guanylate kinase inverted 1 (MAGI-1) in the pathogenesis of proteinuria. In this paper, research progress in the pathogenesis of glomerular proteinuria in recent years is reviewed.

## 2. The Structure of the Glomerular Filtration Barrier

The glomerular filtration barrier is comprised of three layers. (1) Capillary endothelial cells in the inner layer. A variety of fenestrae, small ostioles of 50–100 nm in diameter on endothelial cells, may prevent hematocytes from passing through. These fenestrae may not intercept plasma proteins to be filtrated, but the negative proteoglycans on their surface may exert some charge barrier effect. (2) The cellular basement membrane in the interface layer. This is the major filtration barrier of the filtration membrane, is approximately 100 nm thick, and consists of the inner and outer loose tectoria and the intermediate stratum compactum. The GBM is a microfibrous reticulum structure composed of hydrated gel that exerts its charge-barrier and size-barrier functions via its rich surface anion charges and fibrous strap meshwork-like aperture screen stencil. (3) The visceral epithelial cells in the outer layer. Epithelial cells have podocytic processes that form fissures among them through reciprocal overlapping. There is a layer of filtration fissure membrane on the fissures with holes of 4–14 nm in diameter on its surface that represent the last barrier of the filtration membrane. Under normal circumstances, the radius of an effective filtration hole in the glomerular filtration membrane is approximately 30 angstroms. Small molecular proteins such as lysozyme and *β*2-microglobulin can be filtered, while proteins with molecular weights above 60,000–70,000 are difficult to filter. The molecular weight of plasma albumin is 69,000, and the molecular radius is approximately 37 angstroms, making it difficult to pass through the filtration membrane. Pathologically, as a result of antibasement membrane antibody and immune complex deposition, as well as the release of cytokines and inflammatory mediators, glomerular capillary damage may occur with increased permeability. This allows a large number of plasma proteins, especially albumins in the Bowman's capsule, to exceed the proximal renal tubule's ability to reabsorb proteins and induce proteinuria.

## 3. Glomerular Endothelial Cells and Proteinuria

The difference between glomerular endothelial cells and other vascular endothelial cells lies in their flatter surfaces and fenestrae that are approximately 50–100 nm in diameter. Glomerular endothelial cells are the first line of defense of the glomerular filtration barrier. The fibrils, which are 7 nm thick and exist inside the endothelial cytoplasm, constitute the fenestrate structure. Changes in the aperture of fibrils may affect vessel wall permeability. Endothelial cell structural proteins (e.g., actin, myoglobulin) may also affect the diameter of the fenestrate structure via growth factors. In addition, synergism between the inherent special structures (such as cell membrane-like depression, zonula occludens, and glycocalyx) of glomerular endothelial cells and circulatory permeability factors (such as a-acidic mucin, apolipoprotein, and Amadori's product) may generate the endothelial cell-GBM-podocyte axis, thus playing a special role in maintaining the integrity of the filtration barrier [6, 7].

The cell coat on the surface of glomerular endothelial cells, also known as glycocalyx, is approximately 300 nm thick. It consists of proteoglycan, glycosaminoglycan, and plasma proteins such as orosomucoids that are rich in negative charges. Plasma proteins can be secreted by endothelial cells and are closely related to the permeability of glomerular capillaries. Glycocalyx can affect blood flow velocity and exert a selective barrier effect on macromolecular movement. Damage to systemic endothelial glycocalyx is associated with the onset of albuminuria in patients with type 1 diabetes [8]. Glycocalyx is perturbed in individuals with type 2 diabetes, and oral glycocalyx precursor treatment improves glycocalyx properties [9]. Recently, it has been reported that damage to the endothelial glycocalyx alters the permeability of multiple capillary beds: in the glomerulus this is clinically apparent as albuminuria [10]. It has been found in endothelial cells cultured in vitro that the glycosaminoglycan hyaluronan in glycocalyx may generate a matrix with molecular sieve properties in order to maintain the charge barrier [11, 12]. In addition, orosomucoid plays an important role in maintaining the charge barrier by interacting with the glycocalyx of endothelial cells [6, 13].

## 4. GBM and Proteinuria

Like the basement membranes of other structures of the human body, the GBM is a microfibrous reticular structure primarily consisting of collagen type IV, laminin (primarily laminin-11 and *α*5*β*2*γ*11), nidogen (entactin/nidogen), and heparan sulfate (primarily perlecan and agrin). It is a layer of acellular basement membrane that is 300–350 nm in thickness and plays a role in glomerular filtration as well as podocyte adhesion, migration, and differentiation. Such a molecular structure endows the GBM with the dual functions of being the mechanical barrier and charge barrier, and abnormalities in each role may lead to proteinuria.

### 4.1. Collagen Type IV and Proteinuria

Collagen type IV is a triple helix protein composed of three *α* chains. Its molecular weight is 180 kDa, and it consists of isomeric chains (*α*1–*α*6) encoded by six different genes. These genes form the reticular structure via intermolecular interactions, shaping the fundamental skeleton of the GBM; other molecules attach to it in different ways. During human fetation, collagen type IV is dominated by *α*1.*α*1.*α*2 tripolymer-originating meshwork in the earliest stage of forming the GBM vascular loop; however, with the gradual development and maturity of the glomerular capillary loop, collagen type IV is gradually replaced by *α*3.*α*4.*α*5 tripolymer-originating meshwork. The alteration in collagen type IV during fetation is thought to be related to oxidative and physical stress. In the kidneys, since plasma proteins contain a variety of proteases that contact the GBM directly and the *α*3.*α*4.*α*5 tripolymer is rich in disulfide bonds, the *α*1.*α*1.*α*2 tripolymer may be more resistant to the effects of proteases and various physical stimuli. When gene mutations occur in the *α*1-*α*5 chain, the GBM develops irregular pachynsis, multiple stratifications, and a reticular structure. This is manifested as hereditary nephropathy-Alport syndrome, which is clinically characterized by progressive hematuria, proteinuria, and renal failure. If the gene encoding *α*4 chain is mutated, “thin basement membrane disease” can occur, which is characterized by hematuria under the microscope and is also known as “benign familial hematuria.” When autologous antibodies are present in the NCI structural region of the anticollagen type IV *α*3 chain, the GBM mechanical barrier is disrupted and produces massive proteinuria. This is clinically referred to as “Goodpasture syndrome” [14–16].

### 4.2. Laminin and Proteinuria

Laminin, secondary only to collagen in GBM content, is a heterotrimeric glycosidoprotein composed of *α*, *β*, and *γ* chains. It is cross-shaped and provides the supporting structure for other parts of the GBM. It is believed that there are five *α* chains, three *β* chains and two *γ* chains in humans, forming 11 different types of laminin tripolymer. Mutations in the LAMB2 gene, encoding the laminin *β*2 chain, are associated with the Pierson's syndrome. The human LAMB2 gene maps to chromosome band 3p21 and is composed of 32 densely packed exons spanning about 12 kb of genomic DNA [17]. Mutations in the LAMB2 were also found in patients with congenital nephrotic syndrome [18], and LAMB2 mutations were reported to result in loss of laminin-*β*2 expression in the kidney [19]. LAMB2 knockout mice, which model is Pierson syndrome, show congenital albuminuria followed by podocyte foot process effacement, and they die at about 3 weeks of age with severe neuromuscular defects and nephrotic syndrome [20]. A recent case reported a minor variant of Pierson syndrome in a teenage girl with severe myopia since early infancy and proteinuria first detected at age 6. At the age of 11 she was found to carry a unique homozygous nontruncating LAMB2 mutation [21].

Similar to collagen type IV, during the development of the kidney, a series of changes occur to the upper laminin layer of the GBM, from laminin-10 (*α*5*β*1*γ*1) in the fetal period to laminin-11 (*α*5*β*2*γ*1) in the mature period. Laminin-11, which contains *β*2, is a glycosidoprotein that is indispensable for maintaining the function of the GBM. Although *β*2 knockout mice have an intact GBM ultrastructure, when GBM functions are severely damaged, the mice develop massive proteinuria seven days following birth and extensive pathologically fusion of glomerular podocytic processes occur, similar to the minute lesions in human beings. In addition, the emergence of proteinuria occurs in advance of subpodocytic fusion and SD disappearance, and the mice die 3–5 weeks after birth. This indicates that laminin may play an important role in cell-matrix interaction [22]. Laminin interacts with the various components of the GBM such as agrin, nidogen, and perlecan and is associated with cell surface receptors such as integrin *α*3*β*1, *α*6*β*1 and *α*-dystrophin, which constitutes the molecular biological basis for laminin's participation in cell-matrix interactions [22].

### 4.3. Nidogen and Proteinuria

Nidogen, also known as entactin, is a single-stranded glycosidoprotein of 150 kDa in molecular weight. It was originally extracted from the extracellular matrix of an Engelbreth-Holm-Swarm (EHS) tumor. Nidogen consists of three spherical regions (G1, G2, and G3), and region G3 associates noncovalently with the *γ*1 chain of laminin in an equimolar fashion. Region G2 associates with collagen type IV in a manner similar to how the collagen stroma is linked with the laminin meshwork. This region also binds to basement membrane proteoglycans. Therefore, through its ability to crosslink other GBM components, nidogen plays an important role in the development and maintenance of GBM integrity.

### 4.4. Heparan Sulfate and Proteinuria

Proteoglycans on the GBM are dominated by heparan sulfate (HS) polysaccharide. This includes proteoglycans such as perlecan and agrin, which are distributed in the GBM. Heparan sulfate is rich in negative charges that can limit the ability of negatively charged plasma proteins to pass. It is therefore an important participant in the glomerular charge barrier. HS may also interact with collagen and laminin in the GBM to maintain the structural integrity of GBM molecules. It may therefore also exert an effect as a mechanical barrier [23]. It has been found in animal experiments that one-off intravenous injection of HS monoclonal antibodies or digestion of HS with heparinase in rats may both induce massive proteinuria in rats. Data from perlecan N-terminal gene-knockout mice has shown that there are no obvious morphological changes to the kidneys of these mice under physiological conditions, but their kidneys are highly sensitive to protein load. When injecting bovine serum albumin into these mice, massive proteinuria emerges [23]. In addition, studies on agrin show that it may promote the anions binding to the GBM. These studies show that perlecan and agrin play important roles in maintaining the GBM charge barrier and mechanical barrier.

## 5. Podocytes and Proteinuria

In podocytes and podocytic processes, SDs are important components of the glomerular filtration barrier. Slit diaphragms are zipper-like membranaceous electrodense structures that are linked in a zigzag fashion among podocytic processes. Most studies show that SDs have a rigid structure with a relatively constant diameter (20–50 nm) and are composed of multiple protein complexes. The bridging adjacent podocytic processes are the last barriers of glomerular filtration. The slit membrane can be divided into three parts: an extracellular part (the extracellular portion of the transmembrane protein on the slit membrane), a transmembrane part (the intramembrane part of the transmembrane protein and the membrane-associated protein on the podocytic membrane), and an intracellular part (the cytoplasmic domain of the transmembrane protein, and other membrane-related proteins and slit membrane proteins in podocyte). A number of SD molecules expressed by podocytes have recently been discovered and can be classified into four categories: SD membrane proteins including Nephrin, podocyte skelemins including *α*-actinin-4, basement membrane-podocyte junctional membrane proteins including *α*3*β*1 integrin and podocyte terminal membrane proteins including Podocalyxin [24]. In depth study of the structural molecules of podocytes may accelerate further understanding of the structure and function of the glomerular filtration barrier and may clarify the pathogenesis of proteinuria.

### 5.1. Nephrin and Proteinuria

Nephrin was the first SD molecule found in podocytes. It is the transmembrane protein encoded by the NPHS1 gene and is composed of 1241 amino acids. It belongs to the immunoglobulin superfamily of cell adhesion molecules and is located specifically in the glomerular slit diaphragm region. The extracellular amino terminal region of nephrin molecules has eight Ig repetitive sequences, one interval region and one type III fibronectin-like region. Each Ig motif contains two cysteines (Cys), which may form a disulfide bond in the Ig repetitive structure, endowing the Ig motif with a spherical or elliptical form. If the Ig motif forms a chain-like structure, the slit diaphragm is the widest part of the structure (35–45 nm). In addition to the two Cys residues contained in each Ig motif, the nephrin molecule also has three free Cys: one located in the first Ig motif, one in the interval region and one in the fibronectin-like region. A Cys in the first Ig motif forms a disulfide bond with another Cys in the interval region of the nephrin molecule; the two molecules show homophilic adhesion. In this way, many nephrin molecules form axial filaments along the slit diaphragm. In addition, a Cys in the fibronectin-like region may form a disulfide bond with another nephrin molecule or with an unknown protein connecting the slit diaphragm and the cytoskeleton. In summary, the three free Cys residues participate in the formation of intermolecular disulfide bonds that enhance the integrity of the slit diaphragm. Deletion of these three Cys residues may relax the slit diaphragm, leading to its disappearance, disrupting the filtration barrier and inducing proteinuria [25].

A gene mutation in nephrin can lead to congenital nephrotic syndrome of the Finnish type (CNF), with clinical features including massive intrauterine proteinuria, a placental weight exceeding the body weight by 25% at birth and significant edema. This syndrome is progressive, and patients usually die within 2 years after birth. At the first day after intravenous injection of extracellular antinephrin monoclonal antibody (mAb)5-1-6 into rats, massive proteinuria has been shown to emerge and to peak on day five. Studies on models of nephrotoxic serum nephritis, Heymann nephritis, and amino nucleotide nephropathy determined that the expression of nephrin in renal structures of mice was significantly decreased in the model group. Moreover, the mice developed massive proteinuria [25, 26].

### 5.2. Podocin and Proteinuria

Podocin is an integral membrane protein that was detected while studying steroid-resistant congenital nephrotic syndrome using targeted cloning technology. It is encoded by the NPHS2 gene [27]. Podocin is a new member of the stomatin family of lipid raft-associated proteins and consists of 383 amino acids with the molecular weight of 42 kDa and a “hairpin-like” single membrane region. Its N- and C-termini are located in the cytoplasm. Podocin interacts with nephrin and CD2AP via its C-terminus. It plays an important role in maintaining the structure and function of SDs as a supporting protein. Studies in zebra fish found that at 72 and 96 h after fertilization, the expression of glomerular podocin protein decreased or disappeared, leading to abnormalities in slit diaphragms and preventing the formation of normal podocytic processes [27].

NPHS2 gene-encoding podocin knockout mice showed proteinuria prior to birth and died within several days following birth; their pathological manifestation was severe mesentery sclerosis. Extensive subpodocytic fusion and slit membrane disappearance could be observed via electron microscopy. NPHS2 gene mutations have been detected in syndromes such as human congenital familial steroid-resistant nephrotic syndrome and sporadic steroid-resistant nephrotic syndrome [28–30], suggesting that podocin plays an important role in maintaining the structure of podocytic processes and the integrity of slit membranes. Podocin could be of great importance in the pathogenesis of proteinuria.

### 5.3. CD2AP and Proteinuria

CD2AP is a transmembrane protein belonging to the immunoglobulin superfamily and is composed of 639 amino acids with a molecular weight of 80 kDa. It was initially determined that, as an intracellular ligand of T cell and natural killer cell CD2 receptors, CD2AP can stabilize connections between T cells and antigen-presenting cells. Furthermore, CD2AP is extensively expressed in various structures of humans and mice, and it has been shown through immunoelectron microscopy that CD2AP localizes near the intracellular segment of nephrin in the podocyte side wall. Simultaneous immunofluorescence confirmed that CD2AP is primarily expressed in the glomerulus of the kidney and located at the podocyte slit membrane within the glomerulus and can also be detected in the concentrated pipe as well as some proximal and distal tubules. Its N-terminus contains a Src homology 3 (SH3) domain that can identify the proline-rich amino acid sequence in the intracellular C-terminus of CD2. The middle segment is a proline-rich structural domain that has a variety of loci and can bind with a number of enzymes and protein molecules. The C-terminus has the binding sites for the helical structure region and the actin cytoskeleton; the leucine zipper domain in the C-terminus can regulate the development of the CD2AP homodimer [31, 32]. As an adaptin, CD2AP can interact with slit membrane proteins such as podocin and nephrin via its C-terminus, locating its anchor within subpodocytic lipid rafts in order to preserve the functions of the cytoskeleton and SD. Damage to CD2AP not only affects the function of SDs, but also directly damages the podocyte cytoskeleton, disrupting the stability of the cytoskeleton and leading to pathological changes such as subpodocytic deformation and disappearance, thus inducing massive proteinuria [33]. In addition, CD2AP can interact with various signaling molecules via its SH3 region and participate in cytoskeletal assembly. The CD2AP gene-knockout mouse develops subpodocytic fusion or disappearance one week after birth, mesangial cell proliferation, extracellular matrix deposition and proteinuria at week two, and nephrotic syndrome at weeks three or four and dies of proteinuria and renal failure as a result of subpodocytic defects at weeks six or seven. This indicates that CD2AP is of great importance for maintaining the structure of podocytes and SDs [31, 32]. Nevertheless, no nephrin abnormalities were detected in this model at the genetic level or with respect to protein localization, suggesting that the interaction between CD2AP and nephrin is not necessary for the generation and aggregation of nephrin. Only when severe glomerular injury occurs can changes be observed in nephrin, which might be related to the disappearance of the subpodocytic slit diaphragm. Another study showed that nephrin was expressed earlier than CD2AP and that CD2AP gene-knockout mice showed a gradual disappearance of podocytic processes that were previously normal in appearance. These findings suggest that nephrin can induce podocytic processes independent of CD2AP, but CD2AP is of great significance for maintenance of the morphology and function of the subpodocytic slit diaphragm [26].

### 5.4. NEPH1 and Proteinuria

NEPH1 is a transmembrane protein with a molecular weight of 110 kDa. The human Neph1 gene is located in chromosome 1 (1g21-q25), within a known gene domain relating to childhood nephrotic syndrome. The NEPH family contains three members: NEPH1, NEPH2, and NEPH3. They all belong to the immunoglobulin superfamily and have five similar extracellular immunoglobulin-like repetitive sequences, a transmembrane region and an intracellular region composed of 198–235 amino acids. NEPH1 is extensively distributed in many tissues of humans and mice; its expression level is highest in the kidney. Immunohistochemical staining has shown that the molecule is only expressed in podocytes and immunoelectron microscopy confirmed that the molecule is localized to the slit membrane [34, 35]. Neph1 knockout mice demonstrated similar phenotypes to nephrin knockout mice. At 1 week after birth, they were weaker and smaller than normal mice and without edema; however, with the development of the disease, almost all of the mice developed proteinuria to varying degrees and died 3-4 weeks after birth. Electron microscopy showed the existence of subpodocytic fusion, suggesting a significant role for Neph1 in maintaining the integrity of the glomerular filtration barrier [36].

Nephrin and NEPH1 gene deletions can lead to similar phenomena, such as subpodocytic fusion, proteinuria, and high perinatal mortality, suggesting that they may have the same pathological mechanism. Immunocoprecipitation showed that the extracellular segment of nephrin can interact convergently and divergently with the extracellular region of NEPH1 and its own extracellular region, respectively, but that NEPH1 does not interact with its own extracellular region. This indicates that nephrin and NEPH1 form a kind of heterogeneous oligomer receptor complex on the subpodocytic slit diaphragm by cis-trans interactions which participates in the formation of the SD zipper-like structure and maintains the normal structure of podocytes and the integrity of SDs. This interaction is rather complex and involves multiple immunoglobulin-like structural domains of the two molecules [35, 37].

Podocin has a tissue distribution similar to that of the NEPH family. Immunocoprecipitation showed that the intracellular regions of the three members of the NEPH family can all bind to the podocin C-terminus. Podocin can also precipitate endogenous NEPH1 originating from podocytes. The intracellular segments of proteins in the NEPH family all have a highly conserved sequence composed of nine amino acids (KDPTNGYYxV). NEPH1 gene mutation or replacement of the no.7 tyrosine of the conserved sequence with an alanine can block the association of NEPH1 and podocin, confirming that the integrity of this conserved segment is of great importance for the interaction between podocin and NEPH1. The specific mechanism of podocin and Neph1 interaction involves dephosphorylation of tyrosine 637 of NEPH1 via Tec kinase, which promotes the association of NEPHI and the podocin carbon terminus. The interaction between NEPH1 and podocin plays a significant role in the maintenance of podocyte SDs [38].

### 5.5. *α*-Actinin-4 and Proteinuria

Four members of the *α*-actinin family have thus far been identified: *α*-actinin-1 (nonmuscle type), *α*-actinin-2 (muscle type), *α*-actinin-3 (muscle type), and *α*-actinin-4 (nonmuscle type). Only *α*-actinin-4 expression has been found in renal tissues, primarily in podocytes, and its coding gene, ACTN4, is located on chromosome 19q13. *α*-actinin-4 is an actin filament cross-linked protein with a molecular weight of approximately 100 kDa, and it is an antiparallel homodimer and is dumbbell-shaped (width: 4-5 nm, length: 40–50 nm). It consists of three structural domains: the N-terminal CH domain is an actin binding domain (ABD) containing 250 amino acid residues. The C-terminal 150 amino acid residues constitute the CaM domain, containing two “EF hand” repeats. The central *α*-helix repetitive fragment is comprised of the four spectrin repeats from R1-R4, each fragment consisting of 122 amino acid residues forming a rod-like structural domain. Podocyte *α*-actinin-4 is formed through interaction between the two identical peptide chains via the central spectrins. *α*-actinin-4 can regulate actin polymerization and depolymerization. It bundles the loose actin fibers in podocytes into fasciculi with contraction through the ABD structural domain on both ends in order to stabilize podocyte cytoskeletal structures such as actin and actin filaments, maintain the morphology of podocytic processes, and regulate the movement of the cytoskeleton [39, 40]. *α*-actinin-4 is extensively expressed in podocytes. In experimental nephrotic syndrome as well as primary and secondary human glomerular lesions, *α*-actinin-4 is expressed and abnormally localized, along with abnormal expression of other SD-related proteins [41]. Gene mutations in ACTN4 may lead to focal segmental glomerulosclerosis, suggesting that the actin cytoskeleton may affect the structure or function of podocytes and participate in the occurrence and development of proteinuria [42]. *α*-actinin-4 deletion in mice showed progressive proteinuria, and these mice died within a few months of birth. Early electron microscopy results showed regional subpodocytic fusion which later became diffuse fusion, and indicated that the structure of the SDs was severely disrupted [43]. These results indicate that *α*-actinin-4 is of crucial importance for the maintenance of cytoskeleton and SD functions.

### 5.6. *α*3*β*1 Integrin and Proteinuria

Integrins are a class of molecules belonging to the transmembrane glycosidoprotein cell surface receptor family and are heterodimers formed by *α* and *β* subunits through noncovalent bonds. To date, 16 *α* subunits and nine *β* subunits have been identified, which together assemble into at least 19 kinds of integrins. According to differences in *β* subunits, the integrin family can be divided into three subfamilies: *β*1, *β*2, and *β*3. The kidneys are dominated by the *β*1 subfamily, including *α*3*β*1 integrin and others. In kidneys, *α*3*β*1 integrin is highly expressed in podocytic processes along the glomerular basement membrane, and the molecules exist extracellularly in the form of membrane proteins and contain a transmembrane region and an intracellular region. The *α*3 subunit has the molecular weight of 150 kDa and is encoded by the ITGA3 gene located on chromosome 17. It has been found that this subunit contains a site for binding calcium ions. The *β*1 subunit has four Cys-rich repeats whose macrocycles at the terminal extracellular amino acids are reinforced by intrachain disulfide bonds (S-S). There is also an integrin-linked kinase (ILK) on this subunit. When podocytes are injured, the activated ILK phosphorylates the intracellular region of the *β*1 subunit, thereby reducing the binding between *α*3*β*1 integrin and the basement membrane. The globular regions at the terminal amino acids of the integrin *α*3 and *β*1 subunits interact with each other, forming the extracellular ligand-binding site, and bind with the basement membrane laminin, collagen IV and fibronectin at focal contacts. The cytoplasmic domains of the two subunits, however, are comparatively shorter and bind with subpodocytic cytoskeletal secondary filaments mediated by actin auxiliary protein molecules such as Talin, Vinculin, and Paxillin. The formed “basement membrane-integrin-cytoskeleton” structure not only stabilizes the podocyte cytoskeleton, but also initiates the integrin-dependent signaling pathway as a transmembrane information system. This affects cell morphology and the cell cycle, regulates gene expression and cytoskeletal assembly and contraction, and modulates cell proliferation, differentiation, and apoptosis [44, 45]. Animal experiments showed that anti-*α*3*β*1 integrin antibodies can separate podocytic processes from the GBM and induce proteinuria. In human FSGS patients and in puromycin-aminonucleoside- (PAN-) induced nephropathy mouse models, there were significantly downregulation of *α*3*β*1 integrin expression than in the normal control group. Moreover, their expressions were clearly decreased prior to the morphological changes in the podocytes. It is therefore clear that *α*3*β*1 plays an important role in maintaining the normal morphology and functioning of podocytes [46].

### 5.7. TRPC6 and Proteinuria

Transient Receptor Potential Cation channel 6 (TRPC6) is a hexametric transmembrane protein with intracellular N- and C-termini and a pentameric and hexametic transmembrane structure constituting a nonselective cation channel. The TRPC family can be divided into four subgroups according to structural homology and functional specificity: TRPC1, TRPC2, TRPC4/5, and TRPC3/6/7. A large number of TRPC6 proteins are distributed in brain tissues, with some in the lung and ovary. In the vascular system, TRPC6 is distributed in smooth muscle cells and endothelial cells and participates in the regulation of vascular smooth muscle function. TRPC6 is expressed in the glomerulus and renal tubules, but is primarily localized in podocytes. Immunogold labeling has shown that TRPC6 is located in primary and secondary podocytic processes, especially around the SD annex. Immunofluorescence double labeling showed that podocyte TRPC6 is colocalized with Nephrin, Podocin, and CD2AP, and immunoprecipitation showed that TRPC6 interacted with Nephrin and Podocin, but not with CD2AP [47, 48].

TRPC6 knockout mice primarily exhibited an elevation in blood pressure and increased arterial ring contraction induced by the agonist, indicating that TRPC6 plays a rather important role in regulating vascular smooth muscle function [49]. A TRPC6 gene mutation may cause familial FSGS, as the mutation detection rate of TRPC6 was 7% in familial FSGS. A major clinical manifestation of proteinuria has also been observed. The majority of patients develop end-stage renal diseases about 10 years after onset, and pathological renal manifestations are common in FSGS. Studies on secondary FSGS found that TRPC6-originating calcium influx leads to the abnormal localization of Nephrin in the SD, such that Nephrin is unable to function normally, leading to changes in TRPC6-mediated calcium currents, which are critical in the regulation of intracellular molecules and cytoskeletal behavior in podocytes [49, 50].

### 5.8. Megalin/gp330 and Proteinuria

Megalin/glycosidoprotein (gp330) is a receptor involved in multiple-ligand-mediated endocytosis. It is located on one side of podocytes, as well as in microvilli of clathrin-coated fovea and proximal convoluted tubules. It belongs to the LDL receptor family, and its ligands include apoE-rich *β*-VLDL, lipoprotein (a), lactoferrin, oproteinlipase, aprotinin, plasminogen and others. Under normal conditions, megalin on podocytes can bind with proteins filtered from the GBM and degrade them via endocytosis. Heymann nephritis is an experimental model of human membranous nephropathy. In this model, the following reactions are triggered after binding occurs between a megalin antibody and megalin: (1) an antigen antibody complex-activating alexin cascade, resulting in the formation of C5b-9 membrane attack complex and exerting cytolytic toxicity; (2) enhanced expression of subsolvent NADPH oxidases in podocytes, which are activated and translocated to the cell membrane, generating a large number of reactive oxygen species; and (3) apoE and apoB100 aggregation that is observed at sites of regional aggregation and regional immune complex deposition of various megalin ligands, which are all megalin ligands. apoE and apoB100 undergo peroxidizing modifications as a result of the effects of reactive oxygen species produced during the cytotoxic process initiated by alexin. Lipid peroxidation leads to glomerular capillary wall injury, causing proteinuria that is clear alleviated after treatment with probucol, an inhibitor of lipid peroxidation [51].

### 5.9. WT1 and Proteinuria

Another well-described genetic defect in patients with primary nephrotic syndrome is the spectrum of clinical pictures caused by mutations in Wilms tumor suppressor gene 1 (WT1), a transcription factor regulating the expression of many genes through DNA binding [52]. WT1 was identified by positional cloning in children with the WAGR syndrome, a syndrome characterized by the association of Wilms' tumor (W), aniridia (A), genitourinary malformations (G), and mental retardation (R) [53]. The WT1 gene contains 10 exons and spans approximately 50 kb on chromosome 11. It generates a 3 kb mRNA and encodes a 52–54 kDa protein [54]. In addition to being a tumor suppressor gene, WT1 has been shown to play crucial roles during embryogenesis, especially during kidney development [55]. WT1 mutant mice do not form kidneys and mice lacking the transcriptionally active WT1 splice variant WT1-KTS develop kidneys with very few immature glomeruli [56]. The WT1 gene is widely expressed in epithelial cells of early nephron and is restricted to podocytes in the mature glomeruli [57]. Based on this, WT1 is often utilized as a molecular marker for evaluating podocyte number and density under different circumstances [58]. Several lines of evidence suggest that WT1 may indeed play an important role in the maintenance of normal podocyte function [55]. Heterozygous de novo mutations in WT1 cause Denys-Drash syndrome (DDS) and Frasier syndrome (FS) [59]. WT1 is mutated in 94% of all Denys-Drash syndrome (DDS) patients, companied with the development of glomerular nephropathy involving glomerulosclerosis [55]. WT1 mutations have also been found in patients with nephrotic syndrome and isolated cases of glomerulosclerosis [57, 60]. In addition, WT1 is downregulated in a variety of glomerular diseases with podocyte injury, and WT1 mRNA is detected in the urine of some patients with glomerular diseases [61]. WT1 plays a fundamental role in controlling the expression of major podocyte-specific genes such as nephrin and podocalyxin in adult kidney [62, 63]. Although it has been implicated that changes in the expression of TGF-*β*1, PDGF-*α*, and Pax-2 which are regulated by WT1 affect cytoskeletal architecture [64], the complete set of WT1's targets in podocytes remains to be defined.

### 5.10. PLCE1 and Proteinuria

PLCE1 (phospholipase C epsilon-1) gene locates at chromosome 10q23.32-q24.1, and its encoded protein-phospholipase C*ε*1 (PLC*ε*1) is a member of phospholipase C (PLC) family [65]. PLC*ε*1 is a phospholipase enzyme that catalyzes the hydrolysis of phosphatidylinositol-4,5-bisphosphate and generates two second messengers: inositol 1,4,5-triphosphate (IP3) and diacylglycerol (DAG), which then initiate a cascade of intracellular responses that result in differential gene expression, cell growth, and differentiation [58]. PLC*ε*1 expresses in the matured podocyte of renal glomerulus and plays an essential role in the formation and normal development of capillary loop of glomerulus [65]. The role of PLCE1 in renal pathophysiology remains complicated. Mutations in PLCE1, which was identified as a new cause of autosomal recessive nephritic syndrome in children that present with diffuse mesangial sclerosis (DMS) and FSGS, cause arrest of glomerular podocyte development at the S-shaped stage, thereby halting glomerular development and causing nephrotic syndrome [65, 66]. But enhanced signalling through a form of PLC within podocytes results in podocyte injury and proteinuria [67]. It has been shown that PLC*ε*1 interacted with H-Ras, IQGAP1 (IQ motif-containing GTPase-activating protein 1) and BRAF (v-raf murine sarcoma viral oncogene homolog B1), then serving as crucial intermediates in many signaling pathways [68]. Identification of additional proteins that are expressed in the podocyte and interact directly or indirectly with PLC*ε*1 will be needed to help in the understanding of how mutations in PLCE1 cause nephrotic syndrome.

### 5.11. Lmx1b and Proteinuria

Lmx1b is one of a family of more than nine LIM-homeodomain genes regulating gene transcription via its interactions with gene promoter and enhancer sequences, in conjunction with other transcription factors [69]. Mutations in Lmx1b cause nail-patella syndrome (NPS), an autosomal dominant disease with skeletal abnormalities, nail hypoplasia, and nephropathy [70]. Renal involvement occurs in 25% to 60% of cases, ranging from nonnephrotic proteinuria to end-stage renal disease [71]. Ultrastructurally, foot process effacement was observed for a certain percentage of podocytes [72]. It has been reported that Lmx1b is required for normal podocyte differentiation [73]. Lmx1b −/− mice exhibit kidney defects as well as patterning defects in appendicular skeletal structures and associated soft tissues, and they die shortly after birth [74]. It has been demonstrated that the transcription of podocin is mainly regulated by the transcription factor Lmx1b, which binds to a FLAT-F element and displays enhancer function [75]. However, a study of a podocyte-specific Lmx1b knockout showed later development of proteinuria and greater expression of type IV collagen chains and podocin [76].

### 5.12. SMARCAL and Proteinuria

Mutations in SMARCAL1 (SWI/SNF-related, matrix-associated, actindependent regulator of chromatin, subfamily a-like 1) are involved in the development of Schimke immunoosseous dysplasia (SIOD). This autosomal recessive disorder is characterized by the autosomal recessive transmission of spondyloepiphyseal dysplasia and characteristic dysmorphic features, lymphocytopenia and/or T-cell immunodeficiency, and renal dysfunction including proteinuria and nephrotic syndrome due to FSGS [77]. Two families have been reported in which siblings of affected individuals have incomplete penetrance of SIOD [78, 79]. Furthermore, mutations in SMARCAL1 were also found in two siblings with an incomplete phenotype of SIOD. The siblings were initially classified as suffering from familial steroid-resistant nephrotic syndrome [80]. As SMARCAL1 encodes a SWI/SF2-related protein involved in chromatin remodeling [81], it is tempting to speculate that SMARCAL1 regulates expression of podocyte proteins. However, podocyte genes potentially regulated by SMARCAL remain to be identified.

### 5.13. MYH9 and Proteinuria

An exceptional example of the genetic complexity of nephrotic syndrome was shown by two independent studies demonstrating a strong association of common genetic variants in the MYH9 gene with FSGS and hypertensive ESKD [82, 83]. MYH9 encodes for the heavy chain of nonmuscle myosinIIA (NMMHC-IIA). MYH9 is abundantly expressed in glomeruli, and mainly in podocytes [84]. It has been reported that NMMHC-A acts as a component of the podocyte cytoskeleton, contributing to its contractile functions [85]. A growing body of evidence indicates that loss of MYH9 function may be sufficient to cause kidney disease. Podocyte-specific deletion of MYH9 mice is predisposed to Adriamycin-induced glomerular injury, including podocyte effacement, glomerulosclerosis and proteinuria [86]. Recently, it has been demonstrated that podocytes host response to HIV-1 includes downregulation of MYH9 expression, and this downregulation might play a role in the pathogenesis of HIVAN [87]. Furthermore, MYH9 polymorphisms are associated with diabetic nephropathy in European Americans [88]. However, the underlying pathophysiologic events occurring at the chronic kidney disease associated with MYH9 high-risk haplotypes remain unknown.

### 5.14. SCARB2 and Proteinuria

Mutations in the lysosomal membrane protein human scavenger receptor class B, member 2 (SCARB2), have recently been found to cause action myoclonus renal failure syndrome (AMRF) in humans, which is characterized by collapsing FSGS and progressive myoclonic epilepsy [89]. Clinical report of two siblings revealed that AMRF resulted from a mutation in the SCARB2 gene and the renal involvement was due to nephropathy C1q [90]. SCARB2 (Limp-2 in mice) encodes for the ubiquitously expressed lysosomal integral membrane protein type 2 (LIMP-2) mainly found in lysosomes and late endosomes [91]. This LIMP-2 protein has been shown to act as a receptor to bind *β*-glucocerebrosidase which is a lysosomal enzyme deficient in most cases of Gaucher disease [92]. LIMP-2 knockout mice have tubular proteinuria due to an inability to fuse lysosomes with endosomes and degrade reabsorbed proteins [93]. Recently, although novel SCARB2 mutation has been found in AMRF [94], the pathophysiologic events leading to glomerular disease in cases with SCARB2 mutations remain unknown.

### 5.15. Other Podocyte-Related Molecules and Proteinuria

The components of SDs also include many other podocyte-related molecules, such as Densin, which binds to the cytoskeleton to maintain the polarity of podocytes and interacts with podocalyxin, megalin, and *α*-actinin-4 within podocytes. Moreover, Densin participates in the pathogenesis of proteinuria. Glomerular epithelial cell protein 1 (GLEPP-1) is a newly discovered receptor-like membrane protein tyrosine phosphatase (RPTP). In the kidney, it is specifically expressed at the plasma membrane on top of podocytic processes. P-cadherin plays a role in connecting SD structural molecules. Galloway Mowat Syndrome (GMS) is a rare autosomal recessive disorder comprising of nephrotic syndrome with central nervous system involvement [95]. Linkage studies in two Algerian families identified a homozygous mutation in the GMS1 gene [52]. Recent exome sequencing as well as a whole-genome linkage analysis revealed MYO1E mutations in childhood proteinuric disease and FSGS [96]. MYO1E appears to be important for podocyte motility and may also stabilize the podocyte cytoskeleton [97]. In addition, synaptopodin is an actin-associated protein essential for the integrity of the podocyte actin cytoskeleton because synaptopodin-deficient mice display impaired recovery from protamine sulfate-induced foot process effacement and lipopolysaccharide-induced nephrotic syndrome [98]. Besides, recent studies found that the normal expression of CD38 importantly contributes to the differentiation and function of podocytes and the defect of this gene expression may be a critical mechanism inducing EMT and consequently resulting in glomerular injury and sclerosis [99], and CD151 as a crucial modifier of integrin-mediated adhesion of podocytes to the GBM [100]. However, the functions of these molecules remain undefined and require further study.

## 6. Mitochondrial Disorders and Proteinuria

Mitochondrial DNA plays a crucial role in oxidative production of energy. Thus, defects in mitochondrial DNA can affect virtually all organ systems. Mitochondrial DNA mutations have been recently described also in association with kidney disease, mainly focal and segmental glomerulosclerosis [101].

### 6.1. CoQ10 and Proteinuria

Coenzyme Q10 (CoQ10) is a lipophilic molecule that transfers electrons from mitochondrial respiratory chain complexes I and II to complex III [102]. CoQ10 deficiency is associated with a variety of clinical phenotypes, including nephrotic syndrome [103]. COQ2 mutations have been identified in patients presenting with early-onset NS, severe oliguric renal failure and collapsing glomerulopathy [104]. Dietary supplementation with Q10 provides a dramatic rescue of both proteinuria and interstitial nephritis in the interstitial nephritis mice model [105]. Moreover, administration of CoQ10 had significant beneficial effects on albuminuria in the experimental model of type 2 diabetes, db/db mice [106]. Recently, CoQ10 prevented altered mitochondrial function and morphology, glomerular hyperfiltration and proteinuria in db/db mice, highlighting the role of mitochondria in the pathogenesis of diabetic nephropathy and the benefits of preventing increased oxidative stress [107].

### 6.2. tRNA Mutation and Proteinuria

The point mutations in the transfer tRNA Leu(UUR) gene are mainly associated with the mitochondrial encephalomyopathy, lactacidosis and stroke-like episode (MELAS) syndrome [108]. An A to G transition at nucleotide position 3243 in the mitochondrial tRNA Leu(UUR) gene has showed a pathogenic effect in maternally inherited diabetes and deafness and progressive kidney disease [109]. A3243G mutation is also found in patients with FSGS sometimes associated with maternally inherited diabetes and/or sensorineural hearing loss [110]. In a report of a boy with A3243G mutation in the tRNA Leu (UUR) gene, proteinuria was detected at the age of 6 years, including large amounts of low-molecular-weight proteins such as beta(2)- and alpha1-microglobulin [111]. A3243G mutation was also found in the case of a 59-year-old male with a personal and maternal history of diabetes and deafness, who presented with cardiomyopathy and kidney disease [112]. Furthermore, other mutations in mitochondrial tRNA genes were described in patients with mitochondrial cytopathy presenting with FSGS [113, 114].

## 7. Circulating Factors and Proteinuria

### 7.1. Angiotensin II and Proteinuria

Angiotensin II, traditionally playing a central role as a mediator of glomerular hemodynamic adaptation and injury, is now recognized to exert proinflammatory action leading to upregulation of chemokines, adhesion molecules, and other fibrogenic growth factors [115]. Podocytes are a direct target for angiotensin II—mediated injury by altered expression and distribution of podocyte proteins. Rats receiving angiotensin II by minipump developed hypertension in association with proteinuria. Both real-time PCR and quantitative in situ hybridization demonstrated a significant increase in nephrin gene expression in angiotensin II infused animals compared with control animals [116]. Angiotensin II promotes podocyte injury indirectly by increasing calcium influx and production of reactive oxygen species [117]. Angiotensin II is also closely related to vascular endothelial cells. Under physiological conditions, it regulates the development, maturation, and permeability of endothelial cells [118]. Angiotensin I and angiotensin II, both exert their effects via the Tie2 receptor on endothelial cells. The function of angiotensin I lies in stabilizing endothelial cells and preventing inflammatory responses, angiogenesis, and endothelial cell permeability from increasing. On the other hand, angiotensin II primarily exerts its antiangiotensin I effect via binding to the Tie2 receptor.

### 7.2. VEGF and Proteinuria

Vascular endothelial growth factor (VEGF) is a 43–46 kDa glycoprotein that serves as a key survival factor for vascular endothelium [119]. Through binding with the VEGFR on endothelial cells, VEGF regulates angiogenesis and endothelial cell permeability. In kidneys, angiotensin I and VEGFs, secreted by podocytes, bind to the glomerular vascular endothelial cell receptor Tie2 and VEGFR, affecting the phenotype of endothelial cells and the function of their filtration barrier. Circulating physiological levels VEGF is important for the homeostasis of kidney glomerulus. Blocking VEGF signal transduction by anti-VEGF antibody or soluble receptors could lead to proteinuria. An increase in the incidence of proteinuria has been found in patients receiving anti-VEGF antibody treatment. Moreover, neutralizing VEGFs during blood circulation in mice treated with equimolar anti-VEGF antibody or VEGF receptor may induce proteinuria. At this time point, the major lesion is located in endothelial cells, manifested as vacuolar degeneration of endothelial cells and detachment from GBM. These results suggest that VEGFs may play a significant role in the pathogenesis of proteinuria [120].

### 7.3. Other Circulating Factors and Proteinuria

Fibroblast growth factor 21 (FGF21) is a hepatic hormone involved in the regulation of lipid and carbohydrate metabolism. Plasma FGF21 levels are significantly increased with the development of early- to end-stage CKD and are independently associated with renal function and adverse lipid profiles in Chinese population [121]. Furthermore, it has been reported that serum soluble urokinase receptor (suPAR), which is elevated in two-thirds of subjects with primary FSGS, may cause FSGS [122]. A recent study investigated in 48 stage-2-to-4CKD patients showed that circulating endogenous inhibitor of NO synthase asymmetric dimethylarginine (ADMA) emerged as an independent correlate of proteinuria [123]. Previous study from our group found the mineralocorticoid aldosterone infusion in mice could induce urinary protein excretion and podocyte injury [124]. Although increasing evidence determined that circulating factors played a role in proteinuria, the precise nature of these factors and the mechanisms by which they cause renal injury need further studies.

## 8. Signaling Pathways and Proteinuria

### 8.1. mTOR Signaling and Proteinuria

Mammalian target of rapamycin (mTOR) is a highly conserved serine/threonine kinase, which controls cell growth and metabolism in response to nutrients, growth factors, cellular energy, and stress. mTOR inhibitors rapamycin clinically used for immunosuppression after organ transplantation can cause renal deterioration and proteinuria after conversion from calcineurin inhibitors [125]. mTOR is a widely expressed protein that mediates its functions in two complexes, mTOR complex 1 (mTORC1) and mTORC2 [126]. Podocyte-specific mTORC1 activation in diabetic mice recapitulated many diabetic nephropathy features, including podocyte loss, GBM thickening, mesangial expansion, and proteinuria in addition to podocyte loss [127]. Although mTOR activity was increased in both human and animal kidneys with diabetic nephropathy, genetic deletion of mTORC1 in mouse podocytes induced proteinuria and progressive glomerulosclerosis. Furthermore, simultaneous deletion of both mTORC1 and mTORC2 from mouse podocytes aggravated the glomerular lesions. These results revealed the requirement for tightly balanced mTOR activity in podocyte homeostasis [128]. Therefore, it is important to test if reduction of podocyte mTOR activity can be harnessed as a potential therapeutic strategy to treat diabetic nephropathy [129].

### 8.2. Calcium Signaling and Proteinuria

Calcium ions are important mediators of cellular homeostasis owing to their ability to elicit a dynamic, transient, and tightly regulated range of biochemical responses [130]. The increase of the cytosolic Ca^2+^ activity may be an early event in the pathogenesis of protamine sulfate-mediated retraction of podocyte foot processes [131]. Previous studies had established an intimate association between Ca^2+^ influx and the activation of the Rho GTPases, which are cytoskeleton master regulators [132]. Recent study showed that Rho A played an important role in maintaining the integrity of the glomerular filtration barrier under basal conditions, but enhancement of Rho A activity above basal levels promoted podocyte injury [133]. Moreover, activation of the Ca^2+^-dependent phosphatase calcineurin leads to cathepsinL-mediated cleavage of synaptopodin and to proteinuria. The calcineurin inhibitor cyclosporine A (CsA) blocks the calcineurin-mediated dephosphorylation of synaptopodin and protects synaptopodin from cathepsin L-mediated degradation [134]. Recently, inhibition of calcium channels and chelation of extracellular calcium reduced protamine sulfate-induced foot process effacement and albumin leakage in rat model, and calcineurin inhibitors and the cathepsin L inhibitor all inhibited protamine sulfate-mediated barrier changes. All these results suggested calcium signaling played a critical role in the initial stages of glomerular injury and calcium signaling might act through calcineurin- and cathepsin L-dependent manner [135].

## 9. Conclusion

Renal proteinuria is a manifestation of glomerular filtration barrier dysfunction. It is currently under debate as to which layer of the glomerular filtration barrier plays the most important role in the pathogenesis of proteinuria. Moreover, the functions of some SD molecules remain to be elucidated. The studies described here, however, provide clues for future investigations into the pathogenesis of proteinuria, paving the way for and providing new ways think about treatment options for patients with proteinuria-related diseases.
